# Anti-Müllerian Hormone Levels in Preeclampsia: A Systematic Review of the Literature 

**Published:** 2017-12

**Authors:** Vasilios Pergialiotis, Diamanto Koutaki, Evangelos Christopoulos-Timogiannakis, Paraskevi Kotrogianni, Despina N. Perrea, Georgios Daskalakis

**Affiliations:** 1 Laboratory of Experimental Surgery and Surgical Research, Medical School, National and Kapodistrian University of Athens, Athens, Greece; 2 Department of Obstetrics &Gynecology, National and Kapodistrian University of Athens, Alexandra Hospital, Athens, Greece

**Keywords:** Preeclampsia, AMH, Gestational Hypertension, Systematic Review

## Abstract

**Objective:** Serum Anti-Müllerian hormone (AMH) has been implicated in the pathogenesis of cardiovascular disease. Its prognostic value in determining the risk of developing preeclampsia remains, to date, unclear. The purpose of the present systematic review is to accumulate current evidence in this field.

**Materials and methods:** We searched Medline (1966–2017), Scopus (2004–2017), Clinicaltrials.gov (2008–2017), EMBASE (1980-2017), LILACS (1986-2017) and Cochrane Central Register of Controlled Trials CENTRAL (1999-2017) databases.

**Results:** Four studies were included in with a total number of 401 women. Among them 146 had preeclampsia while 232 were recruited as normotensive controls. Current data are suggestive of the potential predictive value of serum AMH as its levels seem to be lower among women that develop preeclampsia. One study reported that women with and AMH value below the 10^th^ percentile of the studied population had a 3.3 increased risk of developing preeclampsia (OR 3.3, 95% CI 1.2–8.7, p = 0.01).

**Conclusion:** Taking in mind these findings, future studies are needed in this field to establish optimal cut-off values and evaluate the specificity and sensitivity of this biomarker during the first trimester of pregnancy.

## Introduction

Pre-eclampsia affects approximately 2%–8% of pregnancies and represents a major cause of morbidity and mortality worldwide (1). Overall, 10%–15% of maternal deaths are directly associated with pre-eclampsia (2). A variety of maternal risk factors have been implicated including multiparity and pre-existing medical conditions such as autoimmune diseases (systemic lupus erythematosus, antiphospholipid syndrome) and cardiovascular history of hypertension, diabetes or preeclampsia (3).Although the underlying mechanisms are still not fully understood, it is believed that the placenta plays a pivotal role in the pathogenesis of the disease. The impaired migration and invasion of trophoblasts in the maternal uterine spiral arteries results in defective placentation. This leads to placental hypo-perfusion and hypoxia, release of inflammatory factors and endothelial dysfunction

that triggers hypertension (4). Several biomarkers have been proposed for the early detection of preeclampsia. These include proteins such aspregnancy-associated plasmaprotein-A (PAPP-A), pentraxin-related protein (PTX3), plasma protein 13 (PP13), P-selectin, placental hormones (hCG, activin, inhibin), as well as placental factors (e.g. sFlt-1, PlGF and sEng) (5). 

Anti-Müllerian hormone (AMH) is a heavily glycosylated glycoprotein produced by reproductive tissues (6). It is used to determine ovarian reserve and is strongly correlated with the number of antral follicles that gradually decline throughout reproductive life (7,8). A recent report suggested that it may be related to all-cause mortality in men, as each unit increase in serum anti-mullerian hormone level was associated with a 13 % lower risk of death (9). In 2013, Dennis et al suggested that AMH is a putative regulator of the cardiovascular system (10).

Koninger et al. reported that AMH levels decrease during pregnancy as a result of the expected ovarian suppression (11). To date, it remains unknown whether the suppression of AMH is correlated with the process of placentation and whether increased levels of AMH could lead to preeclampsia. Even though several observational studies have reported decreased serum AMH levels, there is a lack of consensus in this field. The purpose of the present systematic review is to investigate whether serum AMH levels significantly differ among women that are at risk of developing preeclampsia.

## Materials and methods

***Study design:*** The present study was designed according to the Preferred Reporting Items for Systematic Reviews and Meta-Analyses (PRISMA) guidelines (12). Eligibility criteria were predetermined by the authors. Language or date restrictions were not applied during the literature research to prevent language bias. All observational (both prospective and retrospective) studies that reported levels of anti-Mullerian hormone among patients with hypertensive disorders of pregnancy and/or preeclampsia and healthy pregnant women were included in the present meta- analysis. Case- reports, review articles and animal studies were excluded. Two authors performed the electronic search of articles and tabulated data on structured forms independently. Any discrepancies during data collection, synthesis and statistical analysis were resolved by the consensus of all authors.

***Literature search and data collection:*** We used the Medline (1966–2017), Scopus (2004–2017), Clinicaltrials.gov (2008–2017), EMBASE (1980-2017), LILACS (1986-2017) and Cochrane Central Register of Controlled Trials CENTRAL (1999-2017) databases in our primary search along with the reference lists of electronically retrieved full-text papers. The date of our last search was set at 1 July 2017.

**Figure 1:**
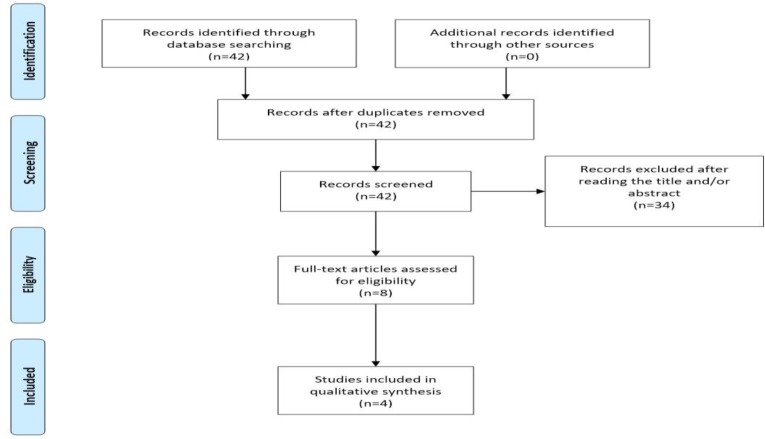
PRISMA flow diagram

Our search strategy included the words “anti-Mullerian hormone, AMH, preeclampsia, gestosis, hypertension” and is schematically presented in the PRISMA flow diagram (Figure 1).

**Figure 2:**
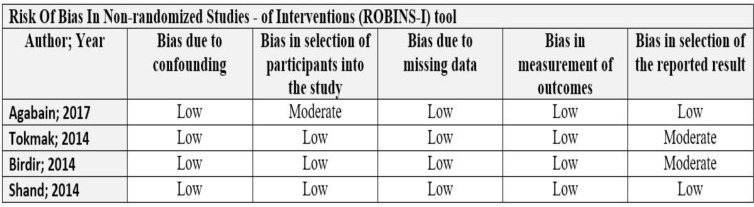
Risk of Bias in non – randomized studies of interventions (ROBINS-I) tool

***Definitions:*** Preeclampsia was defined as the simultaneous presence of hypertension (systolic blood pressure ≥140mmHg or diastolic blood pressure ≥ 90mmHg on at least two consecutive occasions 6 h apart)with proteinuria (1+ in urine dipstick or 300mg per 24-h urine excretion) that first appeared after 20 weeks of gestation in previously, otherwise, healthy women. The definition of preeclampsia as early/late onset was based on the cut-off of34 weeks of gestation. 

***Quality assessment:*** We evaluated the quality of included studies using the Risk Of Bias In Non-randomized Studies (ROBINS-I) assessment tool. The tool briefly assesses the potential confounding, selection, classification, attrition and reporting bias (13) (Figure 2). Studies were assigned a low risk of bias when they were comparable to a well-performed randomized trial with regard to this domain and to moderate risk of bias when they were sound for a non-randomized study with regard to this domain but could not be considered comparable to a well-performed randomized trial.

## Results

***Included studies:*** We finally included 4 studies in our review with a total number of 401 women (14-17). Among them 146 had preeclampsia while 232 were recruited as normotensive controls. In contrast to the other 3 studies, Shand et al enrolled 23 women as controls who had hypertension during their pregnancy (17). The design of each study and the patient eligibility criteria are summarized in Table 1. Table 2 presents the patient characteristics, including the number of patients, maternal age, gestational age, as well as the AMH levels.

***Excluded studies:*** We excluded 4 studies after reading the full text, as they did not meet our inclusion criteria. One of the excluded studies did not disclose the outcomes of interest (18). The remaining investigated the AMH levels after birth (6 months to 10 years later) (19-21). Thus, they were excluded, as their goal was to investigate the effect of preeclampsia on ovarian reserve.

***Outcomes:*** Table 2 summarizes the results concerning the maternal AMH levels of the two groups. Two of the four studies found statistically significant results (p < 0.05) (15, 16), while the results of another study were very close to the level of statistical significance (p = 0.06) (17). More specifically, Tokmak et al and Shand et al report that the maternal AMH levels were elevated in the normotensive group, in comparison to the preeclamptic group. On the contrary, Birdir et al proved the exact opposite correlation. In addition, they conducted analysis of multiples of the median (MoM) for each case and control, to compare more thoroughly the two groups, but found no significant difference (1.040, IQR 0.941–1.081 versus 0.995, IQR 0.939–1.065, p = 0.147) (16).

**Table 1:** Study characteristics

**Author; Date**	**Study type**	**Exclusion criteria**
Agabain; 2017	Case–control	Thyroid disease; hypertension; renal disease, diabetes; liver disease; medication for hypertension; gestational age < 20 weeks
Tokmak; 2014	Case-control	Early onset of preeclampsia; gestational age < 20 weeks
Birdir; 2014	Case-control	Gestational age < 11 or > 13 weeks
Shand; 2014	Retrospective cohort	Multiplepregnancy; birth at < 20 weeks gestation; pregnancy > first trimester

**Table 2:** Patient characteristics (preeclampsia vs control)

**Author; Date**	**Patients number**	**Maternal age (years)**	**Gestational age (weeks)**			**AMH levels**
Agabain; 2017	40 vs 40	27.7 ± 6.9vs 29.5 ± 6.7		36.7 vs 36.9	0.700(0.225–1.500) vs 0.700 (0.400–1.275)ng/ml	
Tokmak; 2014	45 vs 42	28.7 ± 6.2 vs 27.0 ± 4.2		34.0 ± 3.5 vs 38.1 ± 2.6	0.62 ± 0.51 vs 0.93 ± 0.83 ng/ml ¥	
Birdir;2014	50 vs 150	32.6 (29.4–37.1) vs 31.9 (26.9–35.9)		12.6 (12.3–12.9) vs 12.8 (12.3–13.1)	2.140 (1.968–2.273) vs 2.062 (1.938–2.181) ng/L ¥	
Shand; 2014	11 vs 23*	N/A		N/A	4.7 (1.8–13.2) vs 5.5 (1.4–16.1) pmol/L ¥	

* The control group had gestational hypertension;

¥ results were statistically significant (p<.05)

Shand et al reported that pregnant women with lower AMH levels (< 10^th^ centile) in the first trimester of pregnancy had 3.3 times higher chances of diagnosed with gestational hypertension (OR 3.3, 95% CI 1.2–8.7, p = 0.01) (17). Tokmak et al also calculated the receiver operator characteristics curve (ROC) in order to assess the utility of maternal AMH levels in the prediction of preeclampsia. The ROC curve (AUC) was 0.590 (95% CI: 0.469–0.710) (p: 0.149) with a cut-off value of 0.365 ng/ml (sensitivity = 67.4%, specificity = 47.1%) (15).

## Discussion

Our systematic review suggests that higher AMH levels are suggestive of a lower risk of developing preeclampsia during the pregnancy course. To date, it remains unknown whether AMH exerts a protective action in the endothelium or if its value is only prognostic. Nevertheless, the potential prognostic value of AMH for the detection of preeclampsia is also supported by studies that suggest that preeclampsia itself can significantly alter ovarian reserve status and function and thus, decrease AMH levels (19, 21).

AMH has a known contributing role to the evaluation of ovarian reserve. It prevails against other hormonal markers in determining the antralfollical count (8,7) and it is also correlated with fertility (22). Its potential cardio protective and beneficial value in the endothelium is under investigation the last decade. Higher serum AMH levels of female premenopausal cynomolgus macaques decrease the risk of Atherosclerosis and correlate negatively with plaque area (23). Dennis et al reports a negative correlation between AMH and infrarenal aortic diameter (10). In elderly men, higher AMH levels are associated with lower prevalence of Cardiovascular Disease (CVD) (24).On the other hand, increased expression of Pregnancy-associated plasma protein A (PAPP-A), which indicates impaired placental function, seems to enhance the AMH levels in the first trimester of pregnancy (25). Thus, a defective placentation may increase AMH levels and lead to preeclampsia (16). AMH is also elevated in women with PCOS (26), which predisposes gestational hypertension, among other adverse outcomes (27). Moreover, Figueroa-Vega et al observed that log AMH in postmenopausal women was positively related with carotid intima media thickness, a biomarker for predicting early stages of atherosclerosis (28); hence, it is obvious the pathophysiological pattern of this disease and its relation with AMH is not entirely clarified.

***Limitations of our study:*** Our study is the first systematic review in the literature which evaluated the utility of AMH as a predictive factor of preeclampsia. Nevertheless, the relatively limited number of included studies limits safe interpretation of their findings. Three of the four studies evaluated the levels of AMH in the first trimester of pregnancy and only two of them suggested that it may be used as an early marker for the disease. However, only one defined a potential cut-off value and reported the sensitivity and specificity of this biomarker (15).

***Implications for further research:*** Current evidence suggest that serum AMH is implicated in vascular disease. However, its implementation as a screening marker for preeclampsia remains poorly investigated and further studies are needed to reach firm conclusions. These should report outcomes in selected patients, after stratifying them according to the pregestational levels of AMH, to pregnancies that were conceived spontaneously or with ART and according to the severity and onset of the disease. Moreover, sequential measurements should be used, to observe whether serum AMH is altered during the course of pregnancy (a finding that does not seem to be pathophysiologically supported). Future studies should also investigate cut-off values both prespecified and optimal ones to evaluate the sensitivity and specificity of this biomarker.

## Conclusion

Current evidence suggests that higher levels of serum AMH are suggestive of a lower risk of developing preeclampsia. This negative correlation is pathophysiologically supported by clinical and experimental models that seem to implicate low AMH with cardiovascular diseases. However, current evidence remains extremely limited in the field of preeclampsia and future studies are needed to reach firm conclusions.
